# Experimental Traumatic Brain Injury Identifies Distinct Early and Late Phase Axonal Conduction Deficits of White Matter Pathophysiology, and Reveals Intervening Recovery

**DOI:** 10.1523/JNEUROSCI.0819-18.2018

**Published:** 2018-10-10

**Authors:** Christina M. Marion, Kryslaine L. Radomski, Nathan P. Cramer, Zygmunt Galdzicki, Regina C. Armstrong

**Affiliations:** ^1^Center for Neuroscience and Regenerative Medicine,; ^2^Department of Anatomy, Physiology and Genetics, and; ^3^Program in Neuroscience, F. Edward Hebert School of Medicine, Uniformed Services University of the Health Sciences, Bethesda, Maryland 20814

**Keywords:** axon damage, CLARITY, myelin, nerve conduction, node of Ranvier, paranode

## Abstract

Traumatic brain injury (TBI) patients often exhibit slowed information processing speed that can underlie diverse symptoms. Processing speed depends on neural circuit function at synapses, in the soma, and along axons. Long axons in white matter (WM) tracts are particularly vulnerable to TBI. We hypothesized that disrupted axon–myelin interactions that slow or block action potential conduction in WM tracts may contribute to slowed processing speed after TBI. Concussive TBI in male/female mice was used to produce traumatic axonal injury in the corpus callosum (CC), similar to WM pathology in human TBI cases. Compound action potential velocity was slowed along myelinated axons at 3 d after TBI with partial recovery by 2 weeks, suggesting early demyelination followed by remyelination. Ultrastructurally, dispersed demyelinated axons and disorganized myelin attachment to axons at paranodes were apparent within CC regions exhibiting traumatic axonal injury. Action potential conduction is exquisitely sensitive to paranode abnormalities. Molecular identification of paranodes and nodes of Ranvier detected asymmetrical paranode pairs and abnormal heminodes after TBI. Fluorescent labeling of oligodendrocyte progenitors in *NG2CreER;mTmG* mice showed increased synthesis of new membranes extended along axons to paranodes, indicating remyelination after TBI. At later times after TBI, an overall loss of conducting axons was observed at 6 weeks followed by CC atrophy at 8 weeks. These studies identify a progression of both myelinated axon conduction deficits and axon–myelin pathology in the CC, implicating WM injury in impaired information processing at early and late phases after TBI. Furthermore, the intervening recovery reveals a potential therapeutic window.

**SIGNIFICANCE STATEMENT** Traumatic brain injury (TBI) is a major global health concern. Across the spectrum of TBI severities, impaired information processing can contribute to diverse functional deficits that underlie persistent symptoms. We used experimental TBI to exploit technical advantages in mice while modeling traumatic axonal injury in white matter tracts, which is a key pathological feature of human TBI. A combination of approaches revealed slowed and failed signal conduction along with damage to the structure and molecular composition of myelinated axons in the white matter after TBI. An early regenerative response was not sustained yet reveals a potential time window for intervention. These insights into white matter abnormalities underlying axon conduction deficits can inform strategies to improve treatment options for TBI patients.

## Introduction

Traumatic brain injury (TBI) is a major global health concern (Maas et al., 2015). TBI ranging from mild to moderate and severe can result in persistent symptoms (McMahon et al., 2014). Speed of information processing is often slowed in patients across the spectrum of TBI severities and can contribute to slowed reaction times, poor attention and learning, impaired executive function, and ineffective emotional adjustment (Madigan et al., 2000; O'Jile et al., 2006; Willmott et al., 2009; Spitz et al., 2013; Donders and Strong, 2015; Dymowski et al., 2015).

The pathophysiology underlying information processing speed deficits after TBI is unknown. In severe TBI patients, information processing speed is related to diffuse axonal injury and MRI measures of white matter (WM) integrity (Felmingham et al., 2004; Kourtidou et al., 2013). Dysfunction involving the neuron cell body, axon, or synapse can impair information processing and desynchronize neural circuits. Of these circuit components, long axons traversing the WM are particularly susceptible to the compression, torsion, and tension forces that cause axonal cytoskeleton breakdown followed by secondary neurodegeneration within WM tracts (Johnson et al., 2013; Smith et al., 2013).

Our studies focused on analysis of the corpus callosum (CC), which frequently exhibits abnormalities in TBI patients (Rutgers et al., 2008; Shenton et al., 2012; Johnson et al., 2013; S. Chung et al., 2018). Importantly, cognitive impairment has been demonstrated in TBI patients with CC atrophy (Niogi et al., 2008; Wu et al., 2010; Hayes et al., 2016). However, disrupted axon–myelin interactions that contribute to impaired WM function or atrophy are not well understood in TBI patients, or even in animal models. Focal areas of axon and myelin loss can result from TBI that produces vascular damage and hemorrhages because blood vessels are thought to be more vulnerable to TBI forces than axons (Blumbergs et al., 1995; Armstrong et al., 2016a,b). Differentiating irreversible focal loss of axons and myelin from potentially reversible myelin pathology along intact axons is important for understanding the progression of WM injury after TBI.

In the CC, traumatic axonal injury affects both unmyelinated and myelinated fibers (Buki and Povlishock, 2006). Unmyelinated axons are highly vulnerable to damage from TBI (Reeves et al., 2005, 2012). Myelinated axons also incur damage from TBI, which involves the axons and/or their myelin sheaths (Sullivan et al., 2013; Mierzwa et al., 2015). Myelin sheaths enwrap axons to enable rapid impulse firing and a tenfold increase of action potential conduction velocity (Hartline and Colman, 2007). Myelin is also essential for supporting axonal metabolic functions; losing myelin metabolic support can lead to axonal degeneration (Lee et al., 2012). Studies in diseases of myelin have established that myelin loss (i.e., demyelination) slows action potential conduction and exposes the axon to potential further injury (Waxman, 2006). Furthermore, signal conduction velocity is particularly sensitive to disruption of myelin attachment to axons at paranodal regions, which flank each node of Ranvier between myelin sheaths (Babbs and Shi, 2013; Takagishi et al., 2016; Daneshi Kohan et al., 2018). Prior studies in TBI models have not examined action potential conduction velocity relative to axon–myelin pathology in WM tracts.

This study examined morphological alterations in axon–myelin units relative to functional deficits in action potential conduction to determine the contribution of WM injury to slowed processing speed after TBI. A mouse single impact TBI was used to exploit technical advantages while modeling the WM traumatic axonal injury of human TBI (Sullivan et al., 2013; Mierzwa et al., 2015; Yu et al., 2017). Using a combination of technical approaches revealed that WM structural and molecular abnormalities at paranodes correspond with slowed action potential conduction velocity early after TBI. At late time points after TBI, more axons fail to conduct action potentials across the CC, and the CC degeneration progresses to show overt atrophy. Furthermore, between these distinct early and late stages, we demonstrate partial recovery of action potential conduction and evidence of remyelination.

## Materials and Methods

### 

#### Mice and concussive TBI model

All mice were treated in accordance with guidelines of the Uniformed Services University of the Health Sciences and the National Institutes of Health Guide for the Care and Use of Laboratory Animals. Mice were socially housed in 27 cm × 16.5 cm × 12.5 cm cages (2–5 mice per cage) with enrichment objects and maintained on a standard 12 h cycle of daytime light (6:00–18:00). All procedures took place during the daytime light cycle. The following mouse strains were obtained from The Jackson Laboratory: C57BL/6J (RRID:IMSR_JAX:000664), *ROSA^mT/mG^* (RRID:IMSR_JAX:007676; B6.129(Cg)-*Gt(ROSA)26Sor^tm4(ACTB-tdTomato,-EGFP)Luo^/J*), *NG2CreER* (RRID:IMSR_JAX:008538; B6.Cg-Tg(Cspg4-cre/Esr1*)BAkik/J), and Thy1-YFP-16 (RRID:IMSR_JAX:003709; B6.Cg-Tg(Thy1-YFP)16Jrs/J). C57BL/6 mice were purchased as cohorts and acclimated for at least 3 d before use in experiments. All other mice were bred as in-house colonies to generate the experimental mice. The overall number of mice of each strain was C57BL/6J (*n* = 54), *NG2CreER*; *ROSA^mT/mG^* (*n* = 45; referred to as *NG2CreER;mTmG*), and Thy1-YFP-16 (*n* = 20) (see details in Experimental design and statistical analysis). The TBI procedure followed the protocols as previously described (Mierzwa et al., 2015; Yu et al., 2017). Briefly, mice received TBI or sham procedures at 8–10 weeks of age. Under isoflurane anesthesia, the scalp was incised along the midline to expose the skull and a 3-mm-diameter tip was used to impact the skull at bregma (velocity set at 4.0 m/s; depth of 1.5 mm; dwell time of 100 ms). Sham animals were treated exactly the same for all procedures but did not receive the TBI impact. Depressed skull fracture is a predetermined exclusion criterion that was the basis for omitting 1 mouse. Investigators were blinded to animal group allocation until after data analysis. This model results in axon pathology in the CC and external capsule, which is most extensive at coronal levels aligned with the impact site, and in axons in the overlying cingulum and medial cortex (Sullivan and Armstrong, 2017; Yu et al., 2017).

#### Whole-brain tissue clearing

On day 3 after concussive TBI in *Thy1-YFP-16* mice, axon damage under the impact site was visualized through three dimensions (3D) using whole-brain clearing based on the CLARITY process (K. Chung et al., 2013; Tomer et al., 2014). *Thy1-YFP-16* mice have robust yellow fluorescent protein (YFP) expression in motor and sensory neurons that extends through the axons of the CC and sensitively demonstrates varicosities indicative of axon damage (Xie et al., 2010; Gu et al., 2017). Two male Thy1-YFP-16 mice (yoked littermates run as sham and TBI) were deeply anesthetized and transcardially perfused with 20 ml of ice-cold 0.1 m phosphate buffer followed by ice-cold hydrogel monomer solution (4% acrylamide, 0.05% bisacrylamide, 4% PFA, 0.25% VA044 in distilled water), immediately excised and postfixed for 48 h in 20 ml of hydrogel monomer solution at 4°C. The slightly opened sample tube was then placed in a desiccation chamber filled with nitrogen, placed under vacuum for 10 min, and again filled with nitrogen before the tube was quickly closed. The sample was then transferred to a 37°C water bath for 4 h until hydrogel was set, and the brain was removed from the excess hydrogel. The brain was washed 2× with 0.2 m boric acid buffer >24 h, then transferred into a clearing solution (4% SDS in 0.2 m boric acid) in an electrophoretic tissue clearing chamber for ∼1 month until completely transparent. The pH of the clearing solution and presence of fluorescent labeling were checked weekly during the clearing stage. The brain was washed for 1 d in boric acid buffer and then transferred to a refractive index matching solution (88% w/v histodenz, Millipore, Sigma-Aldrich; D2158), 0.1% Tween 20 (Bio-Rad; 1706531), 0.01% sodium azide (Thermo Fisher Scientific; S2271), 0.02 m phosphate buffer) for 48 h before imaging. The brain was mounted with refractive index matching solution in a customized chamber made from Wellco dishes (Pelco; 14032E120), BluTack reusable putty, and Kwik-Sil silicone adhesive (World Precision Instruments; KWIK-SIL) and imaged with a Clr Plan-Neofluar objective (Carl Zeiss, 20×/1.0 NA, 1.45 nD, 0.03 correction collar with 5.6 mm working distance) on an LSM 7MP two-photon confocal microscope (Carl Zeiss). The yoked sham littermate underwent procedures in parallel to confirm that the clearing process did not alter axonal structure or cause swelling unrelated to the injury. The optical image stacks were stitched together using Zen software (Carl Zeiss, ZEN Digital Imaging for Light Microscopy, RRID:SCR_013672), and 3D reconstructions were generated using arivis software (Vision4D Modular Software, arivis AG).

#### Electrophysiological recording

A total of 28 male C57BL/6J mice were anesthetized with isoflurane, brains dissected and transferred to a Leica Biosystems VT1200 vibratome cutting chamber containing chilled sucrose ACSF (in mm as follows: sucrose 206, KCl 2, CaCl_2_ 1, NaH_2_PO_4_ 1.25, MgSO_4_ 2, MgCl-6H_2_O 2, NaHCO_3_ 26, d-glucose 10, bubbled with a mixture of 95% O_2_/5% CO_2_). Coronal sections, 400 μm in thickness, were taken from the region near bregma and the lateral ventricles and transferred to normal ACSF (in mm as follows: NaCl 126, KCl 3, CaCl_2_ 2, NaH_2_PO_4_ 1.25, MgSO_4_ 2, NaHCO_3_ 26, d-glucose 10, bubbled with a mixture of 95% O_2_/5% CO_2_) at 36°C for 20 min and then to room temperature for 1 h. Recordings were performed in an immersion chamber in flowing room temperature normal ACSF continuously bubbled with 95/5% O_2_/CO_2_. Conduction velocities were measured by moving the borosilicate glass recording electrode filled with ACSF along the length of the CC or anterior commissure and capturing the evoked compound action potential (CAP) using a MultiClamp 700B amplifier (Molecular Devices). The slope of a straight line through the plot of CAP latency versus the interelectrode distance yields conduction velocity.

#### Electron microscopy

Prepared grids from 26 male C57BL/6J mice previously quantified for traumatic axonal injury and demyelination (Mierzwa et al., 2015) were reassessed for nodal and paranodal pathologies.

#### Tissue preparation for immunohistochemistry

Mice were deeply anesthetized and transcardially perfused with 0.1 m phosphate buffer followed by 4% PFA, then postfixed overnight in 4% PFA. Dissected brains were cryoprotected in 30% sucrose at 4°C overnight, embedded in OCT compound (Sakura Finetek), and sectioned coronally at 14 μm thickness on a CM 1900 UV cryostat (Leica Biosystems).

#### Confocal microscopy and image processing for analysis of node-paranode complexes

A total of 18 *Thy1-YFP-16* male mice were used for analysis of nodal and paranodal regions. Tissues were prepared for immunohistochemistry, as detailed above, using *Thy1-YFP-16* tissues with primary mouse anti-Caspr antibody (1:500, University of California–Davis/National Institutes of Health NeuroMab Facility 75–001, RRID:AB_2083496) and rabbit anti-Na_v_1.6 antibody (1:500, Alomone Labs; ASC-009, RRID:AB_2040202). Tissues were then incubated with appropriate Alexa dye-conjugated secondary antibodies (1:300, Jackson ImmunoResearch Laboratories; 715-586-151, RRID:AB_2340858 and 711-606-152, RRID:AB_2340625) and counterstained with DAPI (Sigma-Aldrich; D9542) before mounting with Vectashield (Vector Laboratories; H-1400, RRID:AB_2336787). Images of Thy1-YFP with Caspr and Na_v_1.6 immunolabeling were acquired on a 700 laser scanning confocal microscope (Carl Zeiss) with individual laser lines sequentially collected for each channel (pinhole size set to 1 Airy unit) using a Plan-Apochromat 63×/1.4 oil objective at 1.5× zoom and 12-bit color depths. Images were acquired with a voxel size of 0.09 μm × 0.09 μm × 0.45 μm in generating an optical image stack of 67.7 μm × 67.7 μm × 5 μm using a 0.45 μm *z*-interval separation between image planes. The 3D reconstructions of the confocal image stacks were created using Vision4D Modular Software (arivis AG) to improve signal-to-noise ratio and sharpen immunoreactivity boundaries using Curvature Flow Filter anisotropic denoising algorithm (iterations = 20, time step = 0.125). Reconstructions were then captured as a screenshot to generate 2D images, which were cropped using Photoshop CS6 (Adobe Systems, RRID:SCR_014198) and analyzed using National Institutes of Health ImageJ software (RRID:SCR_003070). Two images per animal were processed from equivalent regions of the medial CC of each hemisphere.

#### Quantification of paranode–node complexes

Paranodal domain organization was assessed in ImageJ by manually drawing a line over the length of each domain: left and right paranodes (Caspr) and the intervening nodal gap delimited by Na_v_1.6 immunoreactivity between Caspr domains. The following measurements were tabulated: length of the “triplet” set summed together (Caspr-nodal gap-Caspr), nodal gap length (between Caspr pair), average paranode length (all Caspr domains), average length difference within paranode pairs (paranodal asymmetry), and total number of heminodes (unpaired Caspr domain) per image *z* stack. The asymmetry index for each paranodal pair was calculated as the difference between the longer and the shorter paranode divided by the sum of the length of the two paired paranodes multiplied by 100 (Saporta et al., 2009).

#### Detection of oligodendrocyte lineage cells and myelin in NG2CreER;mTmG myelin reporter mice

*ROSA^mT/mG^* (RRID:IMSR_JAX:007676) were crossed to *NG2CreER* (RRID:IMSR_JAX:008538) mice, providing 45 *NG2CreER;mTmG* mice that were used in experiments. Mice received 20 mg/ml of tamoxifen (Millipore, Sigma-Aldrich; T5648) via oral gavage at 48 and 72 h after TBI or sham procedure (Mierzwa et al., 2014). Tamoxifen induced *Cre* recombination to remove a stop signal and induce a switch from constitutive tdTomato expression in all membranes to GFP labeling of membranes in NG2-expressing cells. Cycling cells were labeled with 5-ethynyl-2′-deoxyuridine (EdU) (Thermo Fisher Scientific; E10187) in cohorts aged to 7 d, 2 weeks, or 6 weeks. The 7 d cohort mice were given two intraperitoneal injections of EdU (100 mg/kg) at 2 h apart on postprocedure days 3–7 (10 total injections), and were perfused 2 h after the last injection. The 2 and 6 week cohorts were given two intraperitoneal injections of EdU on the 4 d before surgery (8 total injections), with the last dose given ∼22 h before sham/TBI procedure. EdU was detected in tissue sections using a Click-iT Plus EdU kit in the far red channel (Thermo Fisher Scientific; C10637) and counterstained with DAPI.

Immunohistochemistry was used to identify specific cell types and myelin for quantification. Mature oligodendrocytes were immunolabeled with primary mouse monoclonal anti-adenomatous polyposis coli (CC1, 1:100, Millipore, Sigma-Aldrich; OP80, RRID:AB_2057371) and incubated with Cy3-conjugated secondary antibody (1:100, Jackson ImmunoResearch Laboratories; 715-166-150, RRID:AB_2340816). Immature through mature stages of oligodendrocyte lineage cells were immunolabeled with primary rabbit polyclonal anti-oligodendrocyte transcription factor 2 (Olig2, 1:100, Millipore, Sigma-Aldrich; AB9610, RRID:AB_570666) and then incubated with Alexa-555-conjugated secondary antibody (1:100, Thermo Fisher Scientific; A-21428, RRID:AB_2535849). DAPI counterstain (Sigma-Aldrich; D9542) was applied before mounting to detect cell nuclei, which aided in differentiating red immunofluorescence of the nuclear localization of Olig2 and the cell body localization of CC1 as distinct from membrane tdTomato (mT) fluorescence remaining in nonrecombined cells within the tissue. Myelin was immunolabeled with primary mouse monoclonal anti-myelin oligodendrocyte glycoprotein (MOG, 1:20) (Armstrong et al., 2002), followed by incubation with AlexaFluor-647-conjugated secondary antibody (1:300, Jackson ImmunoResearch Laboratories; 715-605-151, RRID:AB_2340863) and counterstaining with DAPI (Sigma-Aldrich; D9542) before mounting with Vectashield (Vector Laboratories; H-1000, RRID:AB_2336789). MOG immunofluorescence was detected using secondary antibodies and filters for the far red channel, which did not collect detectable signal from the red tdTomato (mT) fluorescence.

#### Myelin and CC morphology quantification in myelin reporter mice

Images were acquired on an Olympus IX70 fluorescence microscope using a SPOT RT3 camera (Diagnostic Instruments). Myelination was estimated based on pixel intensity values to determine the immunolabeled pixels above background levels within the CC using MetaMorph (MetaMorph Microscopy Automation and Image Analysis Software, RRID:SCR_002368). Myelin fluorescence thresholding to estimate the myelinated area was performed as previously detailed (Armstrong et al., 2006). The CC ROI extended from the midline laterally to under the peak of the cingulum at coronal levels between 0.5 and −0.5 mm relative to bregma. The CC width was calculated as the average of measurements taken at the midline, ∼200 μm lateral to the midline, under the peak of the cingulum, and over the lateral ventricle using MOG staining or Thy1-YFP-16 YFP fluorescence (Mierzwa et al., 2015). Images of newly synthesized GFP-labeled membranes (mG) images were acquired using filters for GFP fluorescence. Images were also acquired an LSM 700 confocal microscope (Carl Zeiss) to illustrate morphological details.

#### Experimental design and statistical analysis

All data collection and analysis were performed by investigators blinded to the sham/TBI condition. All ROIs used coronal levels under the impact site at bregma. Separate cohorts of mice were required for each of the following experimental procedures.

##### Large-scale imaging.

Two male Thy-YFP-16 littermates were killed 3 d after TBI or sham procedures for qualitative analysis. Two confocal image datasets were acquired from the cleared brain of the TBI mouse to allow for more detailed analysis of the ROI. A large-scale dataset was imaged and digitally stitched to generate a 12 × 16 panel image stack ∼2.64 mm across medial to lateral dimension, 1.3 mm deep superior to inferior, and extending 0.518 mm in the caudal to rostral direction. The second dataset was acquired as a single image stack through the CC and cingulum. Both datasets were reconstructed into 3D volumes in arivis to show the scope of injury, and 2D representative images were generated for figure panels.

##### Electrophysiology.

Male C57BL/6J mice were killed after a sham or TBI procedure with the number of mice per time point and condition as follows: 3 d (*n* = 4 sham, *n* = 4 TBI), 2 weeks (*n* = 5 sham, *n* = 5 TBI), and 6 weeks (*n* = 5 sham, *n* = 5 TBI). Surgeries and *ex vivo* slice analysis were performed in yoked pairs, so that 1 sham and 1 TBI animal were injured and assessed together on each experimental day. Slice recordings were performed by a researcher blinded to the condition of the animals. For each mouse, data were collected from a single 400-μm-thick coronal slice, which included the affected region of the CC and the anterior commissure, a WM region unaffected in this model that served as an internal control. Where indicated, each TBI time point was compared with sham groups, which were averaged across time points.

##### Electron microscopy.

Electron microscopy grids prepared from male C57BL/6J mice were assessed for all examples of paranode regions along the axon within the CC. For qualitative analysis, at least two grids of coronal tissue sections were assessed per mouse, with multiple sections imaged per grid. The number of mice evaluated for each time point and condition was as follows: 3 d (*n* = 3 sham, *n* = 4 TBI), 1 week (*n* = 5 TBI), 2 weeks (*n* = 5 TBI), and 6 weeks (*n* = 4 sham, *n* = 5 TBI). Representative images of the observations were selected for each time point.

##### Paranode complexes.

Male Thy1-YFP-16 mice underwent either TBI or sham surgery and were perfused for analysis at 3 d (*n* = 6 sham, *n* = 6 TBI) or 6 weeks (*n* = 3 sham, *n* = 3 TBI) after procedure. Quantification included two confocal images per mouse from equivalent CC regions in both hemispheres of the same coronal section. From each image *z* stack, 100 paranode-node-paranode triplet sets were measured. All heminodes were counted per *z* stack. As axonal YFP signal is a sensitive indicator of axonal damage, the node-paranode measurements and heminode counts were initially performed without the YFP visible to maintain blinding to the condition. Marked heminodes were later confirmed to have YFP present on both sides of the Nav1.6-labeled node but flanked on only one side by a single Caspr-labeled paranode after careful 3D viewing in arivis Vision 4D to include image rotation, fly-through, and varying channel visibility levels.

##### Fluorescent reporter labeling of cells and myelin membranes.

Cohorts of *NG2CreER;mTmG* littermates received TBI or sham procedures and were killed for tissue analysis at 7 d (sham *n* = 3 male; TBI *n* = 3 male), 2 weeks (sham *n* = 3 male, *n* = 2 female; TBI *n* = 3 male, *n* = 2 female), 4 weeks (sham *n* = 4 male; TBI *n* = 3 male, *n* = 2 female), 6 weeks (sham *n* = 4 male, *n* = 1 female; TBI *n* = 3 male, *n* = 2 female), or 8 weeks (sham *n* = 2 male, *n* = 3 female; TBI *n* = 3 male, *n* = 2 female). Four or more images were analyzed per mouse and included images collected from at least two tissue sections per mouse. The CC ROI extended from the midline laterally to under the peak of the cingulum at coronal levels between 0.5 and −0.5 mm relative to bregma, which includes the anterior commissure inferior to the lateral ventricles. NG2mG fate-labeled cells were counted using both the mG and membrane tdTomato labeling to exclude any NG2mG-labeled cells (potential pericytes) along blood vessels.

##### Statistical analysis.

GraphPad Prism 7.0 software (RRID:SCR_002798) was used to graph and analyze all data. The sample size was predetermined from prior experiments as an *n* of 5 or 6 mice per condition for quantification of CC myelination and atrophy, or defined after a predetermined interim power analysis was performed with three mice per condition. The specific sample size and statistical tests used for each set of data are noted in each figure legend. Unpaired Student's *t* test was used to compare TBI and sham for values analyzed at a single time point. For comparison of the frequency of multiple length distributions, measurements were logarithmically transformed using the Y = Log(Y) formula, and the resulting Gaussian distribution was graphed to demonstrate a log-normal distribution. The mean log lengths for each animal were then averaged and compared between conditions using the unpaired Student's *t* test. Two-way ANOVA was used for comparisons of TBI and matched sham conditions at multiple time points or between cell types with *post hoc* analysis using Sidak's test to determine *p* values with consideration of multiple comparisons. Two-way ANOVA with Dunnett's *post hoc* test was used for comparison of multiple TBI postinjury time points to an averaged sham condition. Cohen's *d* effect size was determined by calculating the mean difference between the sham and TBI groups, then dividing the result by the pooled SD. For simplicity, statistical details are only provided for significant comparisons.

## Results

### Large-scale imaging of CC axon damage after TBI

**Figure 1.**
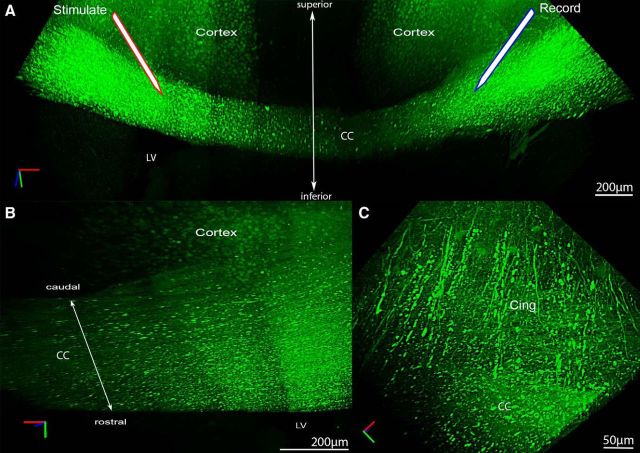
Concussive TBI causes axonal damage in the CC and cingulum under the impact site illustrated using CLARITY. ***A***, Confocal imaging from an optically cleared brain of a Thy1-YFP-16 mouse at 3 d after TBI shows axon damage in the CC, as detected by YFP-labeled axonal swellings. This YFP labeling illustrates the distribution of axon damage in the CC between the positions of the stimulating and recording electrodes used for electrophysiological analysis of axonal conduction properties (Fig. 2). ***B***, YFP-labeled axonal swellings are particularly dense over the lateral ventricle (LV) and extend rostrocaudally throughout the CC under the site of impact. ***C***, Higher magnification of the cingulum (cing) also shows damage in axons with YFP swellings as well as adjacent thinner normal-appearing axons without swellings.

In *Thy1-YFP* mice, YFP-labeled axons exhibit relatively minor variation in diameter with only occasional swellings, as shown in images of CLARITY-cleared specimens of naive (K. Chung et al., 2013) and sham *Thy1-YFP-H* mice, which contrast with enlarged varicosities observed after experimental TBI (Ziogas and Koliatsos, 2018). YFP labeling can reveal axonal swellings from an early reversible stage of damage through to the terminal stage when enlarged swellings disconnect to form axonal end bulbs (Greer et al., 2011; Gu et al., 2017). We have previously shown that axon labeling with YFP in *Thy1-YFP-16* mice detects traumatic axonal injury in the CC after concussive TBI (Yu et al., 2017). Therefore, optical imaging with brain clearing was used for large-scale imaging to detect YFP swellings indicative of axon damage in the CC under the impact site at 3 d after injury (Fig. 1). This large-scale imaging illustrates the distribution of axon damage relative to the ROI in the CC to be used for electrophysiological recordings (Fig. 1*A*). Damaged axons extend across the width of the CC as well as rostrocaudally, and axonal varicosities are particularly localized over the ventricles (Fig. 1*A*,*B*). The TBI also involves axons of the cingulum, where the lower density of YFP-labeled axons allows longitudinal visualization of damaged axons with extensive swellings as well as adjacent large normal-appearing axons (Fig. 1*C*). At this higher magnification, thinner normal-appearing YFP-labeled axons without swellings are visible adjacent to damaged axons (Fig. 1*C*).

### WM conduction deficits after TBI

**Figure 2.**
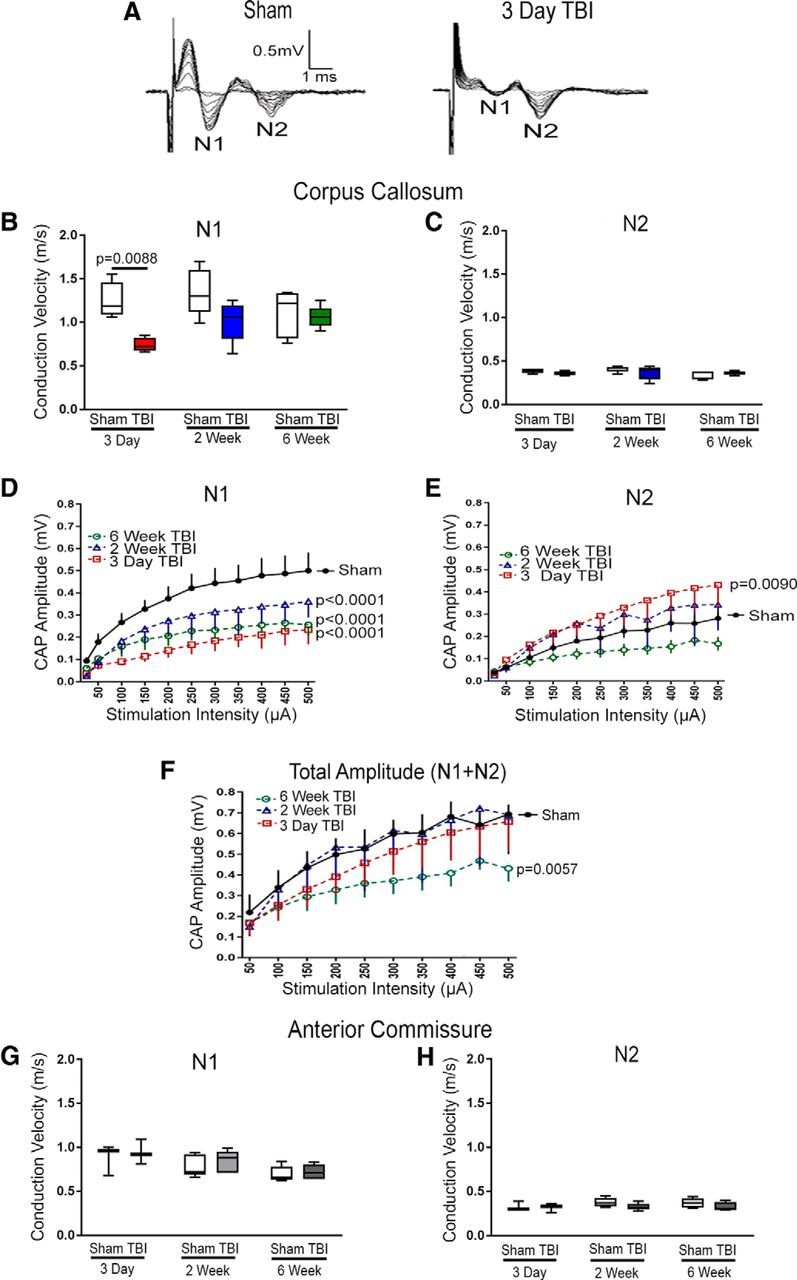
TBI causes slowed conduction velocity followed by axon dropout. ***A***, Representative CAP waveforms recorded from axons within the CC at the level of the midline crossing of the anterior commissure. The fastest wave is the N1 component, which is comprised of myelinated axons. TBI impairs conduction in the N1 fast myelinated axons. The second (N2) wave is comprised of nonmyelinated axons in sham mice but may also include demyelinated axons after TBI. ***B***, TBI slows N1 conduction velocity at 3 d. ***C***, N2 conduction is not slowed by TBI at any time point examined. ***D***, N1 amplitude is reduced at all post-TBI time points relative to the averaged sham values. Among the TBI mice, the N1 amplitude significantly increases between 3 d and 2 weeks (*p* = 0.0003). ***E***, N2 amplitude is increased to above sham levels at 3 d, which may reflect the abnormal contribution of demyelinated axons with conduction velocities that slow to within the timing of the N2 wave. ***F***, Combining N1 and N2 amplitudes shows overall viable axon conduction across the CC and indicates that axon loss and/or conduction block is significant only at 6 weeks after TBI. ***G***, ***H***, The anterior commissure is a WM tract within the same slices served as a technical control for *ex vivo* recording within each brain slice. The anterior commissure is more ventrally located and does not exhibit axon damage in this TBI model. Conduction velocity was not altered in anterior commissure axons in the N1 (***G***) or N2 (***H***) component after TBI, relative to sham mice. Mouse sample sizes were 3 d (*n* = 4 sham, *n* = 4 TBI), 2 weeks (*n* = 5 sham, *n* = 5 TBI), and 6 weeks (*n* = 5 sham, *n* = 5 TBI). Velocities were compared by two-way ANOVA followed by Sidak's multiple comparison test. ***B***, Interaction: *F*_(2,22)_ = 2.7165, *p* = 0.0882; time: *F*_(2,22)_ = 1.7535, *p* = 0.1965; injury: *F*_(1,22)_ = 13.106, *p* = 0.0015 with *post hoc* for 3 d *p* = 0.0088 and effect size = 3.15. Amplitudes compared by two-way ANOVA with Dunnett's multiple comparison to sham. ***D***, Interaction: *F*_(30,138)_ = 0.9489, *p* = 0.9994; intensity: *F*_(10,138)_ = 10.44, *p* < 0.0001; injury: *F*_(3,138)_ = 23.95, *p* < 0.0001 with *post hoc* for 3 d, *p* = 0.0001 and effect size = 1.96, 2 week *p* = 0.0001 and effect size = 0.91, and 6 week *p* = 0.0001 and effect size = 2.01. ***E***, Interaction: *F*_(30,136)_ = 0.3408, *p* = 0.9995; intensity: *F*_(10,136)_ = 6.541, *p* < 0.0001; injury: *F*_(3,136)_ = 10.15, *p* < 0.0001 with *post hoc* for 3 d *p* = 0.009 and effect size = 1.15. ***F***, Interaction: *F*_(27,125)_ = 0.1516, *p* > 0.9999; intensity: *F*_(9,125)_ = 5.661, *p* < 0.0001; injury: *F*_(3,125)_ = 5.31, *p* = 0.0018 with *post hoc* for 6 week *p* = 0.0057 and effect size = 2.49. Error bars indicate 10%–90% interval.

To test the effect of concussive TBI on axonal function in the CC, the CAP velocity and amplitude were evaluated in both sham and TBI mice (Fig. 2). The general placement of the recording and stimulating electrodes in *ex vivo* slices captured signal from axons traversing the CC, including regions exhibiting axon damage (Fig. 1*A*). The CAP has two waveform components: N1 is comprised of fast-conducting myelinated axons and N2 includes slower-conducting axons, which are generally the nonmyelinated axons in healthy adults. Representative waveforms indicate both slowing of the N1 component and loss of N1 amplitude at 3 d after TBI (Fig. 2*A*). Analysis of multiple times after TBI shows that the N1 conduction velocity is slowed significantly at 3 d, followed by recovery (Fig. 2*B*). In contrast, N2 conduction velocity is not significantly different from sham across each after TBI time points (Fig. 2*C*). N1 amplitude is significantly reduced at each time point in TBI mice compared with sham mice (Fig. 2*D*). N2 amplitude increases at 3 d (Fig. 2*E*), indicating that demyelinated axons may have slowed from N1 to fall within the N2 wave. Combining both N1 and N2 amplitudes shows overall axon loss and/or functional dropout is significant only at 6 weeks after TBI (Fig. 2*F*). The anterior commissure, which does not exhibit axon damage in this TBI model, served as a technical control within the same slices. Anterior commissure N1 and N2 conduction velocities were not significantly different from sham mice at any time point (Fig. 2*G*,*H*). Together, these results demonstrate slowed conduction velocity early after TBI followed by dropout of conducting axons during late-phase TBI. In addition, the N1 amplitude increases significantly from 3 d to 2 weeks after TBI (Fig. 2*D*), indicating functional recovery within a time frame expected for myelin repair.

### TBI-induced axon degeneration, demyelination, and node-paranode disruption

**Figure 3.**
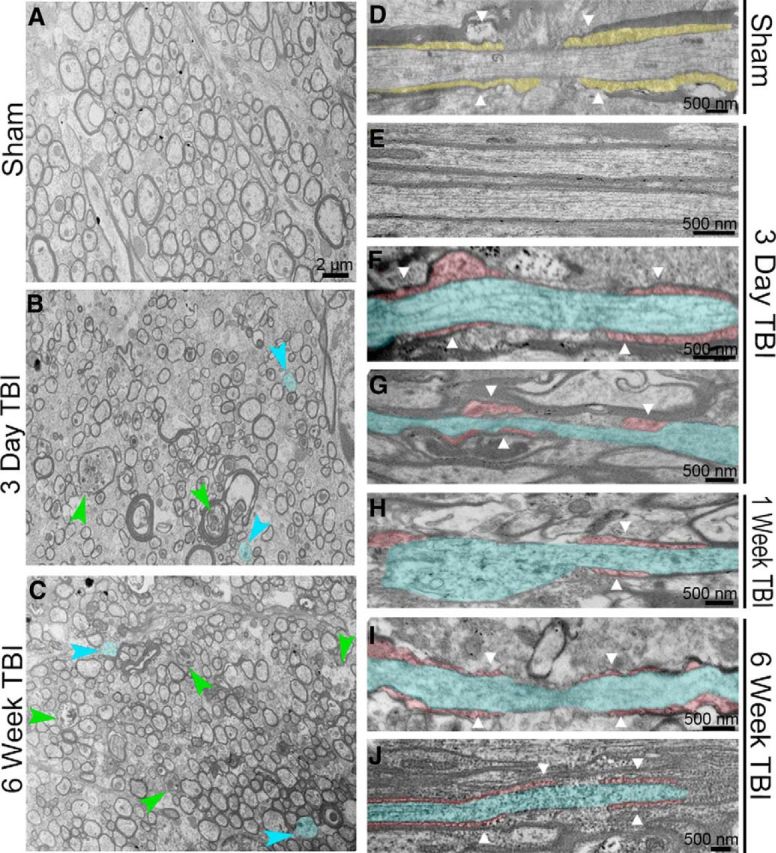
Electron microscopy demonstrates that TBI WM pathology, with characteristic traumatic axonal injury, involves dispersed demyelinated axons and disrupted paranode structure. ***A***, Sagittal sections through the CC show sham axons, many of which are myelinated. ***B***, ***C***, Dispersed degenerating axons (green arrowheads) and demyelinated axons (blue arrowheads and fill) are evident early (***B***) and late (***C***) after TBI. Scale bars, 2 μm. ***D***, Coronal section through the CC to illustrate organized myelin loop attachments forming paranodes (yellow fill; white arrowheads) in sham mice. ***E–G***, Within the CC of injured mice, intact axons with normal myelin (***E***) are found adjacent to damaged axons (***F***, ***G***, blue) with cytoskeletal breakdown and nonuniform diameter, along with abnormal paranodes (red fill) and myelin loss (***F***, ***G***). ***H–J***, Damaged axons (blue) with abnormal paranodes (red) continue to be evident later after TBI.

Rapid action potential conduction is particularly sensitive to disrupted axon–myelin interactions. We used high-resolution electron microscopy to identify a full range of axon and myelin pathology, with a specific focus on nodes of Ranvier and adjacent paranode domains, where myelin attaches to axons. Experimental concussive TBI exhibited dispersed axonal degeneration (Fig. 3), consistent with the pattern of traumatic axonal injury observed in human TBI postmortem specimens (Johnson et al., 2013). Sagittal sections through the CC illustrated healthy axons surrounded by myelin in sham mice (Fig. 3*A*), whereas TBI caused distinct axonal and myelin pathologies at early (Fig. 3*B*) and late postinjury time points (Fig. 3*C*). TBI resulted in both degenerating axons and demyelinated axons (i.e., intact axon without myelin; Fig. 3*B*,*C*) (see also Mierzwa et al., 2015). Coronal CC sections in sham mice have healthy axons with organized myelin loops contacting the axon at paranodal regions flanking the node of Ranvier (Fig. 3*D*). In TBI mice, intact axons demonstrated appropriate axon cytoskeleton and myelin preservation (Fig. 3*E*), whereas adjacent damaged axons showed cytoskeletal breakdown and abnormal paranodal myelin loops (Fig. 3*F*). Early and late phases after TBI exhibited abnormal paranodes with detached, disorganized, or elongated myelin loops associated with damaged axons (Fig. 3*F–J*).

### Abnormal molecular organization of paranodes after TBI

**Figure 4.**
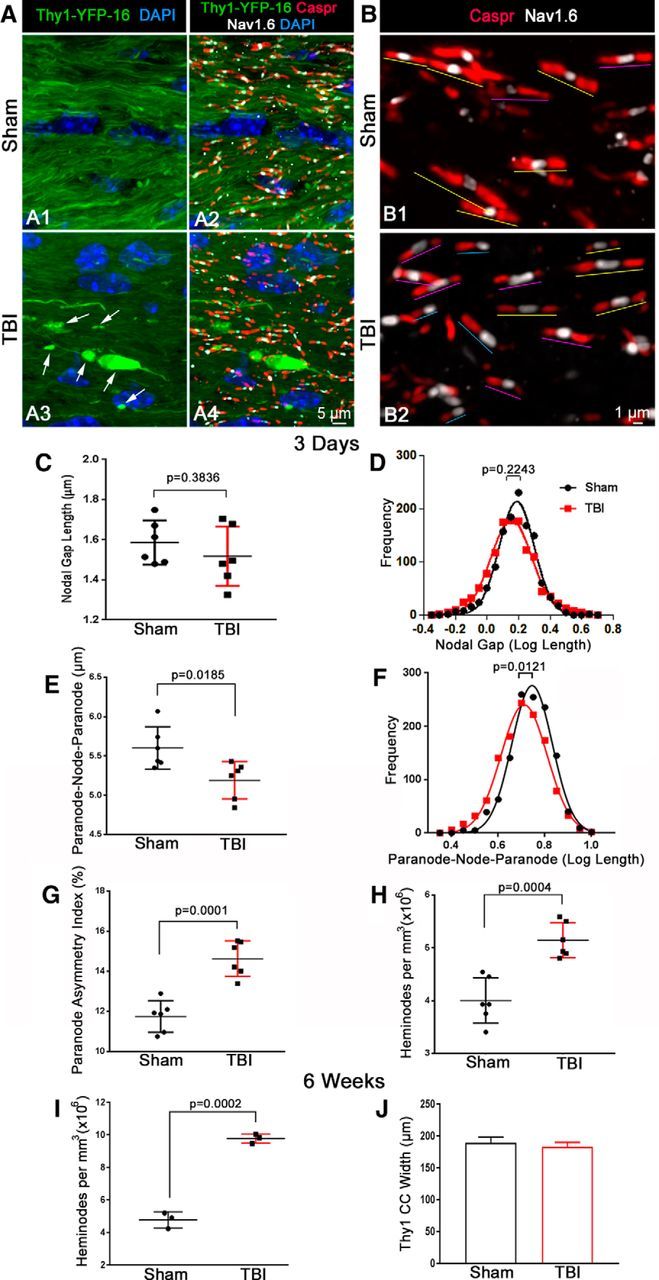
TBI increases paranode asymmetry and formation of heminodes. ***A***, Representative confocal 3D reconstructions of Thy1-YFP-labeled axons in the CC of sham (***A1***, ***A2***) and TBI (***A3***, ***A4***) mice at 3 d after procedure. Thy1-YFP, which accumulates in swellings (arrows) along damaged axons in TBI mice, aided in the analysis of paranodal organization with Na_v_1.6 immunolabeling of nodes of Ranvier (white) and Caspr staining of the flanking paranodes (red). Cell nuclei stained with DAPI (blue). ***B***, Higher-resolution confocal reconstructions show that paranodal complexes in sham animals (***B1***) mostly appear as normal symmetrical units (yellow lines). Following TBI (***B2***), disorganized paranodes are evident as asymmetrical paranodes (purple lines) and heminodes (blue lines). ***C–H***, Quantification of node and paranode parameters at 3 d after sham or TBI procedures. The mean length of the nodal gap between paired Caspr-immunolabeled paranodes remains comparable between sham and TBI animals at 3 d after procedure (***C***). Log transformation plot shows a Gaussian distribution of nodal gap length measurements with no significant differences in mean gap lengths between the two groups (***D***). TBI decreases the overall length of the paranode-nodal gap-paranode regions (***E***). Log transformation plot shows a Gaussian distribution of paranode-nodal gap-paranode “triplet” length measurements with significant shortening in TBI mice (***F***). TBI increases paranodal asymmetry (i.e., shortening of one Caspr-positive paranodal domain in a given paranodal pair) (***G***). TBI also increases the frequency of heminodes (i.e., Caspr domains flanking Na_v_1.6 nodes on only one side resulting in unpaired paranodes) (***H***). ***I***, ***J***, At 6 weeks after procedure, the frequency of heminodes is further increased in TBI mice (***I***), whereas the overall width of the CC is not significantly different (***J***). *p* values were determined by unpaired Student's *t* test. Mouse sample sizes were 3 d (*n* = 6 sham, *n* = 6 TBI) and 6 weeks (*n* = 3 sham, *n* = 3 TBI). ***E***, *t*_(10)_ = 2.18, *p* = 0.0185, effect size = 1.62. ***G***, *t*_(10)_ = 5.973, *p* = 0.0001, effect size = 3.45. ***H***, *t*_(10)_ = 5.158, *p* = 0.0004, effect size = 2.98. ***I***, *t*_(4)_ = 13.71, *p* = 0.0002, effect size = 11.19. ***C***, ***E***, ***G–I***, Error bars indicate 10%–90% interval. ***J***, Error bars indicate SEM.

To further examine these ultrastructural findings of axon–myelin pathology, we used molecular markers to quantify node and paranode domains in Thy1-YFP-16 mice. Three-dimensional reconstructions were used to examine the YFP-labeled axons combined with immunolabeling for Caspr in paranodes and Na_v_1.6 voltage-gated sodium channels in nodes of Ranvier (Fig. 4*A*). Enlarged images of these high-resolution reconstructions enable distinct identification of nodal complexes with symmetrical or asymmetrical paranode pairs as well as heminodes, which have only one Caspr domain adjacent to the Na_v_1.6-immunolabeled node (Fig. 4*B*). TBI did not significantly alter the mean nodal gap length (Fig. 4*C*,*D*). The individual paranodal lengths were also not statistically different between sham and TBI animals (sham = 2.008 ± 0.045, TBI = 1.836 ± 0.065; unpaired *t* test, *p* = 0.0539; mean ± SEM). However, TBI shortened the overall length of the paranode–node-paranode complex (Fig. 4*E*,*F*) and caused asymmetry between paranode pairs (Fig. 4*G*). Importantly, TBI significantly increased the frequency of abnormal heminodes relative to sham mice at 3 d (Fig. 4*H*) and at 6 weeks (Fig. 4*I*). The CC width was not significantly reduced at 6 weeks after TBI (Fig. 4*J*). Therefore, CC atrophy did not account for this increased heminode density between TBI and sham mice. Heminodes were not associated with large-axon swellings along damaged axons in the TBI mice (Fig. 4*A*).

### TBI does not alter oligodendrocyte progenitor density or proliferation in *NG2CreER;mTmG* myelin reporter mice

**Figure 5.**
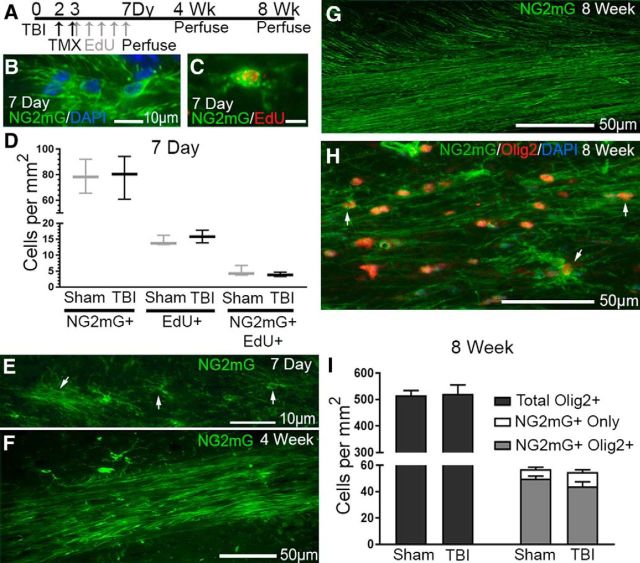
Fluorescent labeling of new membrane synthesis in oligodendrocyte lineage cells using *NG2CreER;mTmG* myelin reporter mice. ***A***, *NG2CreER;mTmG* mice were given tamoxifen on days 2–3 after TBI or sham procedures to induce expression of membrane-localized GFP driven from the NG2 promoter (NG2mG). The thymidine analog EdU was given daily between 3 and 7 d after TBI/sham to label cycling cells during DNA synthesis. ***B–E***, At 7 d after TBI/sham, NG2mG labels cells with a progenitor morphology (***B***), including EdU-labeled cycling cells (***C***). The density of NG2 cells and/or cycling cells in the CC is not significantly different in TBI mice compared with sham (***D***). ***E–G***, NG2mG also labels cells with more elaborate processes that are characteristic of later-stage oligodendrocyte lineage cells (***E***). With longer survival time, NG2mG-labeled cells extend membranes along axons (***F***) that continue to increase within the CC in a myelinating pattern (***G***). All images show representative examples from TBI mice. ***H***, ***I***, The oligodendrocyte lineage marker Olig2 labeled nuclei within the majority of NG2mG cells (***H***, ***I***). At 8 weeks after TBI/sham, the injury condition did not significantly alter the cell populations expressing single or double labeling for Olig2 and/or NG2mG. Mouse sample sizes were 7 d (*n* = 3 TBI mice, *n* = 3 sham mice), 4 weeks (*n* = 5 TBI, *n* = 4 sham), and 8 weeks (*n* = 5 TBI mice, *n* = 5 sham mice). Two-way repeated-measures ANOVA showed no significant effect of injury for a given cell type labeling on *post hoc* analysis with Sidak's adjustment for multiple comparisons. ***D***, Interaction: *F*_(2,8)_ = 0.03029, *p* = 0.9703; cell type labeling: *F*_(2,8)_ = 135, *p* < 0.0001; injury: *F*_(1,4)_ = 0.0002, *p* = 0.9891. Error bars indicate 10%–90% interval. ***I***, Interaction: *F*_(2,27)_ = 0.06392, *p* = 0.9382; cell type labeling: *F*_(2,27)_ = 0.05687, *p* < 0.0001; injury: *F*_(1,27)_ = 0.00617, *p* = 0.9380. Error bars indicate SEM.

In this TBI model, we previously showed that NG2-immunolabeled cells were increased in the dorsolateral extension of subventricular zone at 2 and 6 weeks, compared with sham mice (Mierzwa et al., 2014). Within the CC, NG2 cell density was maintained as well, indicating that genetic fate-labeling of NG2 cells may be feasible for detecting newly synthesized myelin after TBI. Transgenic *NG2CreER;mTmG* myelin reporter mice were used to examine potential changes in immature oligodendrocyte lineage cells and myelin membrane synthesis in the CC after TBI. In these myelin reporter mice, tamoxifen induces recombination to initiate GFP labeling of newly synthesized membranes in NG2-expressing cells (i.e., NG2mG) (Zhu et al., 2011). After TBI or sham procedures, tamoxifen was given on days 2–3 to fate-label NG2 cells and EdU was given on days 3–7 to label cycling cells (Fig. 5*A*). NG2mG fate-labeled cells exhibiting the morphology of oligodendrocyte progenitors were identified at 7 d (Fig. 5*B*). NG2mG-labeled cells located on blood vessels (data not shown) may be pericytes and so were excluded from these counts. NG2mG cells continued to proliferate in the CC (Fig. 5*C*). These NG2 and EdU double-positive cycling oligodendrocyte progenitors comprised 33.65% of the EdU-labeled cells in sham mice and 25.12% after TBI (Fig. 5*D*). However, there was no significant difference in the number of NG2mG-labeled cells, EdU-labeled cells, or NG2 and EdU double-positive cells between TBI and sham mice at 7 d (Fig. 5*D*). In addition, NG2mG fate-labeled cells that had undergone further oligodendrocyte lineage differentiation were observed at 7 d (Fig. 5*E*). At 4 weeks, NG2mG labeling was also present in membranes extending along axons (Fig. 5*F*). At 8 weeks, NG2mG-labeled membranes extended throughout the CC in a pattern consistent with myelin formation (Fig. 5*G*). The majority of NG2mG fate-labeled cell bodies are colabeled with the oligodendrocyte lineage marker Olig2, which was not different between TBI and sham mice (Fig. 5*H*,*I*).

### NG2-mG fate-labeled cycling progenitors and terminal differentiation are not altered by TBI

**Figure 6.**
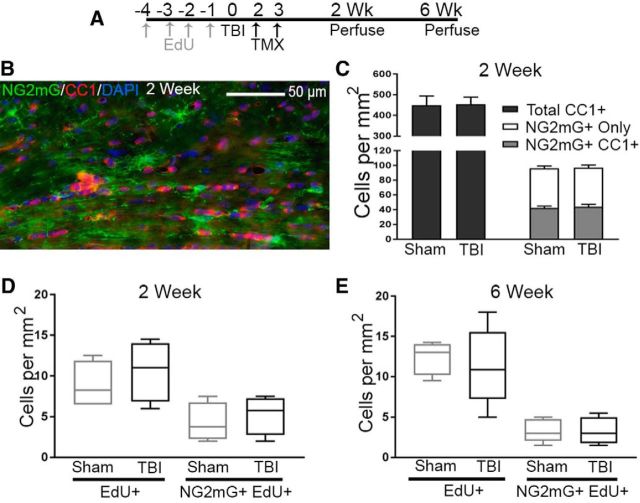
TBI does not alter the response of endogenous cycling cells or differentiation into mature oligodendrocytes in *NG2CreER;mTmG* mice. ***A***, *NG2CreER;mTmG* mice were given thymidine analog EdU daily for 4 d before TBI or sham procedure, followed by tamoxifen on days 2–3 after TBI/sham to induce NG2mG expression. ***B***, ***C***, At 2 weeks after TBI/sham, NG2mG is expressed in immature oligodendrocyte lineage cells and in cells that express CC1, a mature oligodendrocyte marker (***B***). The injury condition did not significantly alter the CC cell populations expressing single or double labeling for CC1 and/or NG2mG (***C***). ***D***, ***E***, The most immature oligodendrocyte lineage cells are endogenous cycling cells that incorporate EdU before TBI/sham procedures. TBI did not alter the population of EdU-labeled cells in the CC, either with or without NG2mG labeling, at 2 weeks (***D***) or 6 weeks (***E***) after TBI/sham. Mouse sample sizes were 2 weeks (*n* = 5 TBI, *n* = 5 sham) and 6 weeks (*n* = 5 TBI, *n* = 5 sham). Two-way repeated-measures ANOVA showed no significant effect of injury for a given cell type on *post hoc* analysis with Sidak's adjustment for multiple comparisons. ***C***, Interaction: *F*_(2,24)_ = 0.005887, *p* = 0.9941; cell type labeling: *F*_(2,24)_ = 202.8, *p* < 0.0001; injury: *F*_(1,24)_ = 0.009432, *p* = 0.9234. Error bars indicate SEM. ***D***, Interaction: *F*_(1,12)_ = 0.0.06658, *p* = 0.8007; cell type labeling: *F*_(1,12)_ = 11.84, *p* = 0.0049; injury: *F*_(1,12)_ = 0.8952, *p* = 0.3627. Error bars indicate 10%–90% interval. ***E***, Interaction: *F*_(1,16)_ = 0.1492, *p* = 0.7403; cell type labeling: *F*_(1,16)_ = 45.24, *p* < 0.0001; injury: *F*_(1,16)_ = 0.1649, *p* = 0.6900. Error bars indicate 10%–90% interval.

To specifically follow endogenous cycling oligodendrocyte progenitors in *NG2CreER;mTmG* mice, EdU was administered daily for 4 d before the TBI or sham procedures, and then tamoxifen was given on days 2–3 after the procedure to fate label NG2 cells (Fig. 6*A*). Approximately half of NG2mG fate-labeled cells underwent terminal differentiation into mature oligodendrocytes by 2 weeks after TBI/sham based on immunolabeling for CC1 (Fig. 6*B*,*C*). The injury condition did not result in a significant difference in the total population of CC1^+^ oligodendrocytes, NG2mG fate-labeled cells, or the subset labeled with both CC1 and NG2mG (Fig. 6*C*). Focusing on the cycling progenitors labeled with EdU before the sham or TBI procedures, there was no significant difference in the total number of EdU^+^ cycling progenitors or the proportion EdU^+^ cells fate-labeled for NG2mG in TBI versus sham mice at 2 weeks (Fig. 6*D*) or 6 weeks (Fig. 6*E*). Together with the postprocedure EdU^+^ labeling (Fig. 5*D*), these data indicate that TBI does not stimulate proliferation to amplify the subset of cycling NG2mG progenitor cells in the CC.

### TBI increases myelin membrane remodeling

**Figure 7.**
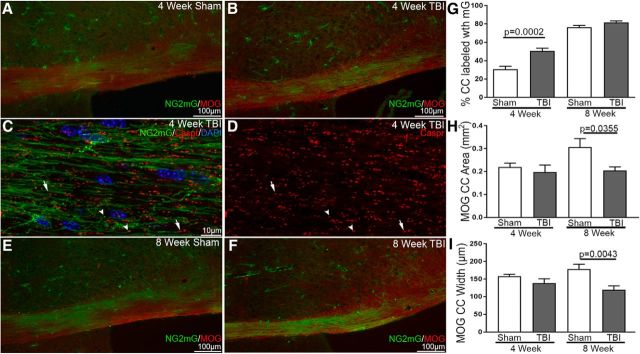
*NG2CreER;mTmG* myelin membrane remodeling and CC atrophy after TBI. ***A***, ***B***, Sham and TBI mice at 4 weeks elaborate NG2mG-labeled membranes in the CC area that is immunolabeled for MOG. ***C***, ***D***, Myelin formation by NG2mG-labeled cells is indicated by membrane extension to paranodes, identified by Caspr immunolabeling. Caspr labels paranode pairs (arrows), as expected for flanking the node of Ranvier. Individual Caspr regions (arrowheads) reveal abnormal paranode organization after TBI. ***E***, ***F***, By 8 weeks after TBI or sham procedures, NG2mG membranes are more widespread within the CC. ***G***, Quantification of NG2mG and MOG shows that TBI increases NG2mG membrane formation in the CC at 4 weeks after injury. ***H***, ***I***, However, at 8 weeks, TBI results in significant CC atrophy. MOG measurements of the area (***H***) and width (***I***) of the CC are reduced in TBI mice, which do not exhibit the normal continued increase with age that is observed in sham mice. Mouse sample sizes were 4 weeks (*n* = 5 TBI, *n* = 4 sham) and 8 weeks (*n* = 5 TBI, *n* = 5 sham). Two-way ANOVA and *post hoc* analysis with Sidak's test for multiple comparisons. ***G***, *F*_(1,15)_ = 8.695, *p* = 0.0100; time: *F*_(1,15)_ = 229.5, *p* < 0.0001; injury: *F*_(1,15)_ = 25.07, *p* = 0.0002 with *post hoc* for 4 weeks, *p* = 0.0002 and effect size = 3.04. ***H***, Interaction: *F*_(1,15)_ = 2.05, *p* = 0.1727; time: *F*_(1,15)_ = 2.781, *p* < 0.1161; injury: *F*_(1,15)_ = 4.908, *p* = 0.0426 with *post hoc* for 8 weeks, *p* = 0.0355 and effect size = 1.89. ***I***, Interaction: *F*_(1,15)_ = 2.887, *p* = 0.1100; time: *F*_(1,15)_ = 0.004485, *p* = 0.9476; injury: *F*_(1,15)_ = 11.42, *p* = 0.0041 with *post hoc* for 8 weeks, *p* = 0.0043 and effect size = 2.14. Error bars indicate SEM.

Following this progression of NG2mG fate-labeled cells toward mature oligodendrocytes, we further examined the extent of NG2mG labeling of myelin membranes. Immunohistochemistry for MOG detected myelin and Caspr identified paranodes in *NG2CreER;mTmG* mice (Fig. 7). At 4 weeks after TBI/sham, NG2mG membranes aligned along axons in a pattern matching with MOG in the CC (Fig. 7*A*,*B*). NG2mG membranes also extended to Caspr-labeled paranodes (Fig. 7*C*,*D*), which is indicative of myelin formation. In addition to the expected paranode pairs, single Caspr domains were observed, in agreement with our finding of increased heminodes after TBI (Fig. 4). At 8 weeks after TBI/sham, NG2mG membranes extended throughout much of the CC (Fig. 7*E*,*F*). Quantification of NG2mG and MOG labeling in the CC shows significant new membrane formation at 4 weeks after TBI (Fig. 7*G*). However, this increase in NG2mG is transient and does not persist at 8 weeks. In sham mice, NG2mG and MOG show continued myelin formation in the adult CC (Fig. 7*E–G*). This result agrees with an increase in the proportion of myelinated axons and number of myelin lamellae in the mouse CC in this time frame (Sturrock, 1980). Importantly, the CC area and width do not increase between 4 and 8 weeks after TBI, resulting in significant CC atrophy relative to sham (Fig. 7*H*,*I*).

## Discussion

Deciphering the diverse pathophysiological mechanisms in play after head injury is important for improving noninvasive biomarkers for TBI diagnosis and prognosis and for developing effective treatments. Experimental TBI in animals enables modeling of specific features of human TBI to examine pathophysiological mechanisms with specialized techniques across early through late postinjury time points. Our studies model traumatic axonal injury in WM tracts, which is a major pathological feature commonly observed in TBI postmortem cases (Smith et al., 2013). The results reported here are the first to show slowed conduction velocity across a WM tract with traumatic axonal injury from TBI. We show that myelinated axons initially exhibit slowed conduction, which recovers over a 2 week period after TBI. This pattern is consistent with disrupted axon–myelin interactions (i.e., demyelination and/or myelin detachment from axons) followed by remyelination. Indeed, this interpretation is strengthened by ultrastructural and molecular pathological evidence of demyelination and disrupted paranode organization. In addition, our studies with *NG2CreER;mTmG* myelin reporter mice provide clear evidence of increased myelin membrane synthesis, which is consistent with remyelination occurring after TBI. However, with longer survival after TBI, our electrophysiological and morphological data show that this WM injury progresses to overall loss of functional axons and CC atrophy. These results demonstrate dynamic structural and functional changes that may contribute to impaired information processing resulting from WM injury in TBI patients.

Neural circuits for sensorimotor, cognitive, and emotional domains depend on myelination to precisely coordinate action potential conduction to synchronize timing and ensure fidelity of transmission (Almeida and Lyons, 2017). Within neural circuits, our studies focused on mechanisms that may impair action potential conduction in injured WM tracts. We focused specifically on myelinated axons because the electrical advantages of myelin enable rapid signal conduction, and transmission along long axons is a major component within neural circuits (Budd and Kisvárday, 2012; Almeida and Lyons, 2017). We show degenerating axons dispersed among intact axons in the CC (Figs. 1, 3), as expected for traumatic axonal injury after TBI (Smith et al., 2013). These same CC regions also contain demyelinated axons (i.e., relatively large axons missing myelin sheaths) (Fig. 3) (Sullivan et al., 2013; Mierzwa et al., 2015). Among the myelinated axons within WM, electron microscopy is required to identify this pattern of demyelinated axons, which is not detected well by immunohistochemistry for myelin proteins (Sullivan et al., 2013; Yu et al., 2017). Similarly, disruption of myelin attachments to axons at paranodal junctions can be widespread without significant overall myelin loss (Takano et al., 2012).

Myelin sheath attachment to axons at paranodes flanking each node of Ranvier is critical for rapid action potential transmission via saltatory conduction (Poliak and Peles, 2003). Prior work in TBI models has shown axonal damage at nodes of Ranvier within 24 h after either experimental stretch injury in guinea pig optic nerve, fluid percussion injury in rats, or lateral head acceleration in nonhuman primates (Maxwell et al., 1993; Maxwell, 1996; Reeves et al., 2010). Our studies demonstrate ultrastructural pathology of nodes at multiple longer survival times along with abnormalities in paranode regions that flank nodes (Fig. 3). We identify increased heminodes at 3 d after TBI (Fig. 4) based on Caspr localization, which molecularly defines paranode regions (Poliak and Peles, 2003). Axonal membrane proteins, such as Caspr and Na_v_1.6, at paranodes and nodes connect within the axons to cytoskeletal proteins, including α-II spectrin and Ankryin-G, respectively. These cytoskeletal proteins can protect axons from mechanical injury and undergo proteolysis after axon damage from TBI (Reeves et al., 2010; Huang et al., 2017). Therefore, damage to nodes and disruption of paranodes are important features of TBI WM injury, in addition to cytoskeletal breakdown and impaired fast axonal transport that produces swellings along the length of myelinated axons (Stone et al., 2001; Greer et al., 2013).

Axonal paranodes have complex molecular interactions to attach myelin and segregate nodal sodium (Na_v_) channels away from juxtaparanodal potassium (K_v_) channels for rapid saltatory conduction and membrane potential repolarization (Poliak and Peles, 2003; Amor et al., 2017). In healthy adult CNS, myelin plasticity adjacent to nodes is a predicted mechanism to precisely time signaling within the CNS (Arancibia-Cárcamo et al., 2017). Both demyelination and myelin detachment at paranodes can slow signal conduction along axons, whereas more extensive myelin or axon damage will typically block signal propagation, resulting in failure of the action potential to reach the synapse (Arancibia-Cárcamo and Attwell, 2014; Freeman et al., 2016; Hamada et al., 2017). Fluid percussion TBI studies in rats have reported reduced CAP amplitude in the first week after TBI (Baker et al., 2002; Reeves et al., 2005) but did not examine conduction velocity or the progression of WM conduction deficits out to the late phase of CC atrophy as in the current study. We show that TBI causes early conduction slowing, which progresses to conduction failure and/or loss of functional axons (Fig. 2), which agrees with the impaired saltatory conduction observed with demyelination and paranode disruption in other neurological diseases (Susuki, 2013; Griggs et al., 2017). Interestingly, computational modeling of TBI also predicts a role for paranode disruption in slowed and/or failed axonal depolarization (Volman and Ng, 2014). After TBI, we found that paranodes were abnormally shortened or missing, which resulted in an increased frequency of heminodes (Fig. 4). This alteration of paranode molecular elements may be an early indicator of myelin damage (Susuki, 2013). Alternatively, asymmetrical paranodes and heminodes may occur where new myelin sheaths are forming, such as during developmental myelination (Freeman et al., 2016; Brivio et al., 2017), or where remyelination is incomplete, as reported in multiple sclerosis lesions (Coman et al., 2006).

Transgenic mice facilitated detection of new myelin formation or remodeling after TBI. *NG2CreER;mTmG* myelin reporter mice given tamoxifen after TBI, or sham procedure, heritably labeled immature oligodendrocyte lineage cells that differentiated into oligodendrocytes and progressively extended membranes along axons (Figs. 5, 6). Caspr immunolabeling colocalized where these fluorescently labeled membranes ended along axons, indicating myelin attachment at paranodes (Fig. 7*C*). This result is consistent with formation of new myelin sheaths and/or new membrane synthesis to remodel existing myelin sheaths. Initial studies using retroviral infection to express *NG2* promoter-driven *Cre* recombinase in *mTmG* mice showed that new fluorescently labeled myelin was formed between 2 and 4 weeks after traumatic spinal cord injury (Powers et al., 2013). This time interval for remyelination is in agreement with our findings in TBI for partial recovery of myelinated axon conduction (Fig. 2*D*) and the transient increase of new membrane formation in our *NG2CreER;mTmG* mice (Fig. 7*G*). Remyelination in the first weeks after TBI is also supported by our ultrastructural finding of thin myelin relative to axon diameter in the CC (Mierzwa et al., 2015). However, our findings also demonstrate CC atrophy and loss of functional axons at longer survival times after TBI (Figs. 2*D*,*F*, 7*H*,*I*). Furthermore, oligodendrocyte lineage cell density (Fig. 5) is maintained during CC atrophy (Fig. 7), indicating a corresponding reduction of axons and the oligodendrocyte population at 8 weeks after TBI. Therefore, interventions that target axon–myelin pathology in the first weeks after TBI may help maintain this remyelination and partial functional recovery to prevent progressive WM degeneration.

These results identify important pathophysiological mechanisms of WM injury after TBI that correspond with symptoms experienced by TBI patients. Further study is warranted using approaches that can extend and address limitations of the current work. Examination of additional molecular components across time after injury is needed to evaluate loss or redistribution of ion channels in the axolemma to more specifically interpret our electrophysiological data. To more directly interrogate the mechanism(s) involved, interventions to modify these pathological features should be tested. In addition, the role of neuroinflammation in these identified functional and structural changes should now be explored. Quantitative *in vivo* electrophysiological approaches are needed to corroborate our *ex vivo* recordings of CC axons. Clinically applicable techniques to detect these pathophysiological changes in TBI patients could be extremely helpful for diagnosis and prognosis. In this TBI model, *in vivo* diffusion tensor MRI detected reduced CC WM integrity (Yu et al., 2017). Advances in diffusion tensor imaging may improve capabilities for quantifying atrophy and loss of axons within an overall volume of WM atrophy (Benjamini et al., 2016). Similarly, demyelination and detached paranodal junctions can be detected by diffusion tensor imaging in simplified models (Xie et al., 2010; Takano et al., 2012).

In conclusion, this study reveals structural, functional, and molecular pathology of axon–myelin interactions in WM after experimental TBI. Furthermore, we demonstrate a progression of WM injury that initially recovers yet advances to later-phase degeneration with CC atrophy. These pathophysiological mechanisms may be responsible for impaired information processing experienced by patients at acute through late phases after TBI. Repetitive TBI may also damage axon–myelin interactions based on myelinated axonopathy observed in humans and slowed conduction velocity reported in mice (Tagge et al., 2018). Importantly, these results inform diverse therapeutic strategies to maintain axon function and promote myelin plasticity, and indicate a potential treatment window in the first weeks before the progression of WM degeneration.
